# Unsymmetrical Diboron Reagents: Application in Borylation Reactions of Unsaturated Bonds

**DOI:** 10.3390/molecules24071325

**Published:** 2019-04-04

**Authors:** Siyi Ding, Liang Xu, Zongcheng Miao

**Affiliations:** 1 Key Laboratory of Organic Polymer Photoelectric Materials, School of Science, Xijing University, Xi’an 710123, China; dingsiyi2009@163.com; 2 School of Chemistry and Chemical Engineering/Key Laboratory for Green Processing of Chemical Engineering of Xinjiang Bingtuan, Shihezi University, Shihezi 832003, China; xuliang4423@shzu.edu.cn

**Keywords:** diboron reagents, borylation reaction, regioselectivity, metal-catalyzed, metal-free, mechanistic study

## Abstract

In the past decades, borylation reactions have received extensive research interest and have developed into effective tools in the synthesis of versatile organoboron compounds. Boranes and symmetrical diboron compounds are commonly utilized as borylating reagents in these transformations, especially in the borylation reactions of unsaturated bonds. More recently, several types of unsymmetrical diboron reagents have been synthesized and applied in these borylation reactions, allowing for complementary chemo- and regioselectivity. This review aimed to highlight the recent development in this rising research field, focusing on new reactivity and selectivity that originates from the use of these unsymmetrical diboron reagents.

## 1. Introduction

Organoboron compounds, especially boronic acids and their derivatives, have served as the preeminent practical building blocks for the construction of molecular complexity and diversity for decades [1]. Due to their versatile reactivity, they play a pivotal role as practical and fundamental platform molecules in synthetic organic chemistry. It has been well documented that they can be used as essential carbon nucleophiles in many types of transformations to introduce functional groups. In addition to various reactions that build up carbon-carbon bonds (e.g., Suzuki–Miyaura coupling) [2], a variety of carbon-heteroatom bonds can be constructed through the transformation of organoboron compounds (e.g., via oxidation and Chan–Lam coupling) [3,4]. This has stimulated the tremendous application of organoboron compounds in the preparation of synthetical intermediates, organic materials, fine chemicals, and drug candidates. Meanwhile, the development of efficient and convenient strategies to introduce boryl groups into readily-obtained organic feedstocks has been persistently pursued [5,6,7,8,9,10,11,12]. Given the versatility of organoboron compounds, increasing their accessibility from other readily available raw materials will not only enrich the library of borylated structural units, but also provide opportunities for rapid diversification of the raw materials.

**Scheme 1:**
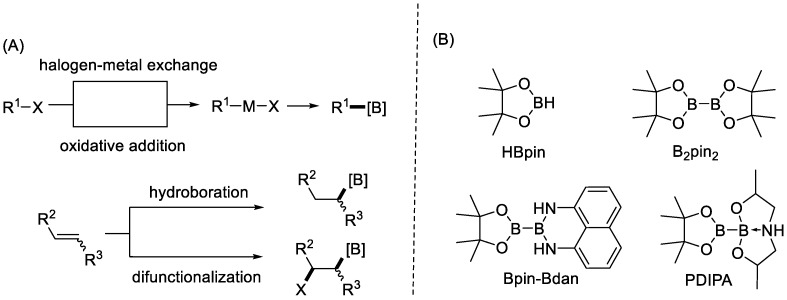
(**A**) Two main pathways for the preparation of organoboron compounds; (**B**) representative borylating reagents.

Typically, to access organic boronic acids or boronates, there are two main pathways (Scheme 1A). One pathway takes advantage of the transformation of organic halides. The other relies on the addition of B–H or B–M (boron–metal) bonds to unsaturated compounds, such as alkenes and alkynes.

Usually starting with the organic halides, highly reactive organometallic intermediates with C–M (carbon–metal) bonds, such as C–Li and C–Mg bonds, are first prepared via halogen–metal exchanges. Then, transmetalation with borates provides borylated products. Alternatively, C–M bonds can also be obtained via oxidative addition processes. In 1995, Pd-catalyzed borylation of aryl halides was achieved by Miyaura et al. with bis(pinacolato)diborane (B_2_pin_2_) for the first time, by using the intermediary C–Pd bonds [13]. Since then, many researchers have been committed to the development of new synthetic routes to construct C–B bonds via similar strategies. The latter protocol has been used more widely than the former organometallic pathway, due to its relatively milder reaction conditions and higher functional group compatibility.

When the starting materials are changed to unsaturated compounds, the addition of B–H bonds of organoboranes can be achieved via stoichiometric [14,15] or catalyzed [16] hydroboration pathways. More recently, many types of difunctionalization reactions [10,11,12] that incorporate boryl groups have also been studied. In these cases, diboron reagents are usually utilized as the boryl source, involving key intermediates that contain B–M bonds [17,18].

Various organoboron compounds can be accessed via the above-mentioned protocols. As for the borylating reagents (Scheme 1B), symmetric diboron reagents, especially B_2_pin_2_, have been utilized most frequently. Recently, unsymmetrical diboron reagents have been developed and applied in many borylation reactions with unsaturated bonds. Interesting chemo- and regioselectivity has been observed in many cases. These advances are highlighted herein, with a focus on clarifying the different selectivity for these unsymmetrical diboron reagents.

## 2. sp^2^-sp^3^ Diboron Reagents

An excellent review article has been published on the preparation, properties, and application of sp^2^-sp^3^ diboron reagents [19]. The following provides a brief summary of these types of reagents, which will not only complement the context of this topic, but will also benefit an understanding of borylation reactions.

Generally, monoquaternization of sp^2^-sp^2^ diboron reagents produces sp^2^-sp^3^ diboron reagents. Such transformations have been explored for a long time since it is not only a research interest of main group chemistry, but also the crucial conversion in the activation of various sp^2^-sp^2^ borylating reagents [20,21]. The latter process is usually achieved via the assistance of additive, single Lewis bases, which produces lengthened, polarized, and more reactive B–B bonds. In the in situ generated sp^2^-sp^3^ diboron reagents, the nucleophilicity of the unquaternized sp^2^ boron atoms is enhanced, which facilitates their transfer to active metal catalysts or organic electrophiles. Therefore, broadly defined, sp^2^-sp^3^ diboron reagents can be seen as the active boryl source in most borylation reactions.

**Scheme 2:**
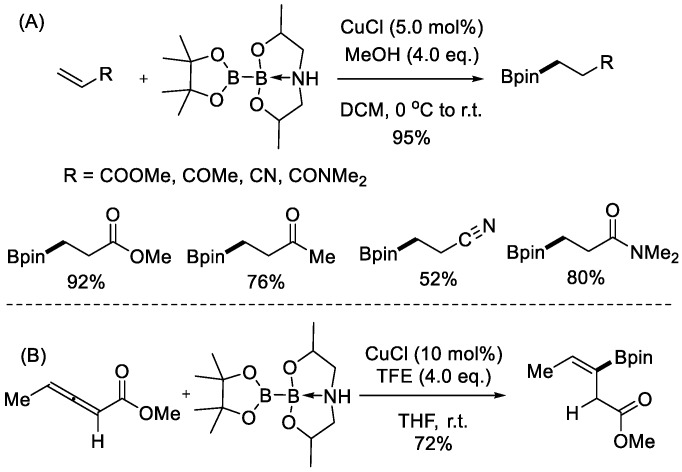
Pinacolato diisopropanolaminato diboron (PDIPA diboron) in copper-catalyzed addition reactions under representative reaction conditions. (**A**) Using α,β-unsaturated conjugated compounds as the starting materials; (**B**) using allenoates as the starting materials.

In 2009, a preactivated sp^2^-sp^3^ diboron reagent, pinacolato diisopropanolaminato diboron (PDIPA diboron), was developed by Santos et al. [22], which contained mixed sp^2^-sp^3^ hybridized boron atoms and a lengthened, active B–B bond. Such a structure removes the requirement for an external base in the boryl transfer process. Santos and Marder et al. [23], applied this preactiviated reagent into the copper-catalyzed β-boration of α,β-unsaturated conjugated compounds, including esters, ketones, nitriles, and amides under ligand and base-free conditions (Scheme 2A). The sp^2^-hybridized boron moiety of PDIPA diboron was selectively transferred to the β-position of electron-poor olefins. Later, the same group completed a copper-catalyzed regio- and stereoselective boration of electron-deficient allenoates [24], thereby installing a boron moiety on the β-position with exclusive (*Z*)-double bond geometry (Scheme 2B). In all of these cases, the Bpin moiety was selectively transferred.

**Scheme 3:**
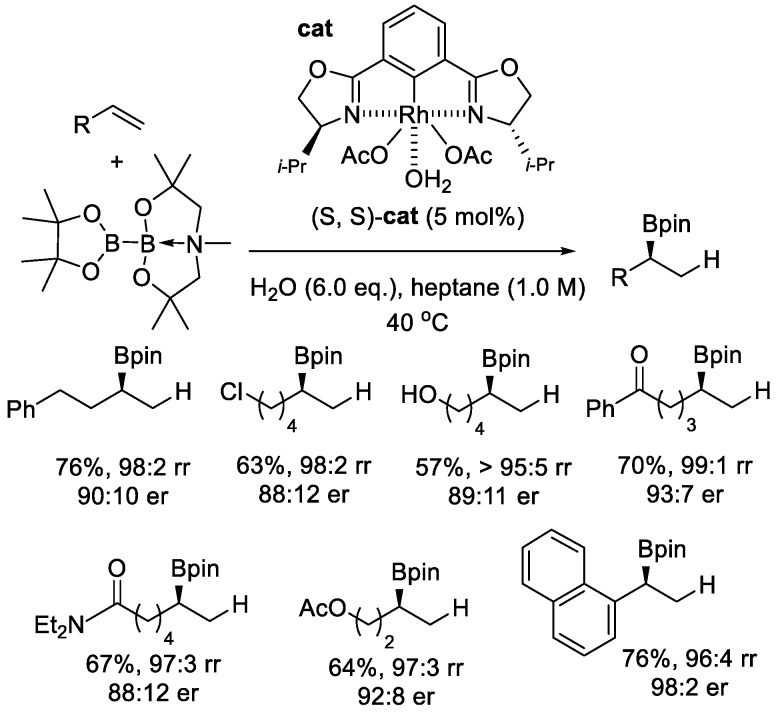
Asymmetric hydroboration of unactivated terminal alkenes.

More recently, taking advantage of the novel sp^2^-sp^3^ hybridized diboron reagent and rhodium catalyst, the Aggarwal’s group achieved the first Markovnikov asymmetric hydroboration of inactivated terminal alkenes (Scheme 2 and Scheme 3) [25]. In this reaction, very high levels of regioselectivity and enantioselectivity could be obtained without the need for directing groups or electronically biased alkenyl substrates. A variety of the functional groups were compatible with the obtained reaction conditions, and many terminal alkenes underwent the hydroboration process easily, except for 1,1-disubstituted alkenes and conjugated diene. The reaction was designed as an interrupted diboration reaction. Commonly, after the insertion of a Rh–B bond into carbon-carbon double bonds, a secondary C–B bond could be introduced via *σ*-bond metathesis between the terminal Rh(III)–alkyl species and an sp^2^-sp^2^ diboron reagent. This process was inhibited when an sp^2^-sp^3^ diboron reagent was used because one of its boron centers was coordinatively saturated, rendering metathesis unfeasible. Instead, a protodemetalation process occurred and created the desired Markovnikov hydroboration products.

## 3. sp^2^-sp^2^ Diboron Reagents

When one of the two same masking groups of symmetrical diboron reagents, such as B_2_pin_2_, is replaced by another bidentate ligand, an unsymmetrical sp^2^-sp^2^ diboron reagent is obtained. In this case, the two boryl moieties of the reagent will be different and, consequently, its applications in different borylation reactions will have to deal with the selectivity problems. These transformations will be discussed according to the specific types of reactions below.

### 3.1. Addition Reactions: Diboration

**Scheme 4:**
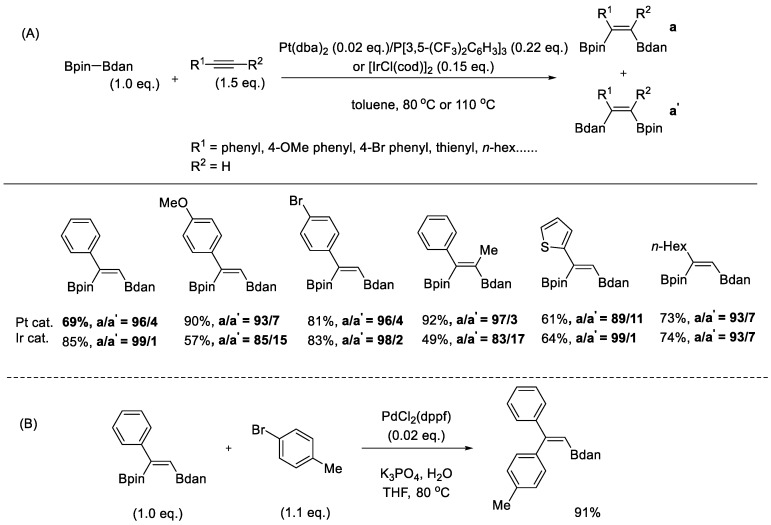
(**A**) Using unsymmetrical diboron reagent Bpin–Bdan in the Ir- or Pt-catalyzed diboration of alkynes with Bpin–Bdan; (**B**) The diborylated compound using in the Suzuki-Miyaura coupling reaction.

The B_sp2_–B_sp2_ hybridized unsymmetrical diboron reagent Bpin–Bdan (pinacolato-1,8-diaminonaphthalenato diboron) was developed by Suginome et al., who applied this reagent into the Ir- or Pt-catalyzed addition reactions of alkynes [26]. This diboration transformation yielded differentiated 1,2-diboronylalkenes, with Bdan groups incorporated into the terminal carbon regioselectivity (Scheme 4A). It is worth mentioning that the regioselectivity of the reactions stayed high, regardless of the variation of the electronic and steric factors in the alkynes. The electron-withdrawing and -donating functional groups on the aryl groups were well-tolerated. The 2-thiophenyl and aliphatic terminal alkynes also resulted in corresponding regioselective products.

In addition, the two incorporated boryl moieties, Bpin and Bdan, could be easily differentiated in the following Suzuki-Miyaura coupling reactions, where Bpin, at the internal position, reacted preferentially (Scheme 4B). Such selectivity was contrary to the situation when both boryl groups were Bpin units.

**Scheme 5:**
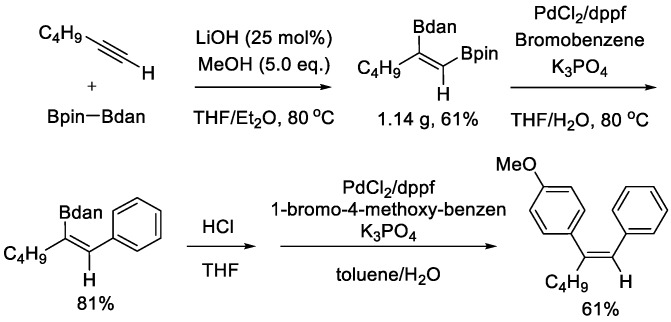
LiOH-catalyzed diboration of alkynes and its application.

During the preparation of this manuscript, Huang, Liu, and Peng disclosed a similar diboration process of terminal alkyl alkynes [27]. In this approach, no noble catalyst was used, and LiOH was utilized as the catalyst. A highly regio- and stereoselectivity was observed. Two boryl groups of Bpin–Bdan were incorporated into carbon-carbon triple bond in *cis* fashion. Unlike the Ir- or Pt-catalyzed approach, the Bdan moiety was incorporated at the internal position. In spite of this difference, the prior reactivity of Bpin in the Suzuki-Miyaura coupling remained unchanged. Trisubstituted alkenes could be obtained via the chemoselective sequential cross-coupling reactions (Scheme 5).

**Scheme 6:**
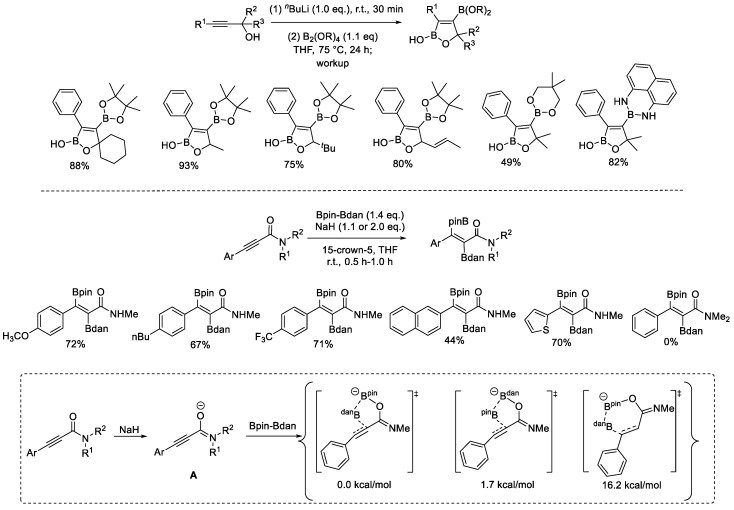
Substrate-assisted diboration of alkynes with Bpin–Bdan.

Uchiyama et al. described an unprecedented *trans*-selective diboration reaction of propargylic alcohols through a *pseudo*-intramolecular reaction [28]. Heteroatoms were installed on the acetylene skeleton to coordinate and activate B–B bonds of diboron reagents, in order to reduce activation energy. The propargyl alkoxides were first prepared by deprotonation between propargyl alcohols and bases. The interaction between the formed anion and a diboron compound allowed the direct diboration of the C-C triple bond without any catalyst, creating a product containing the oxaborole ring unit. When Bpin–Bdan was used as the borylating reagent, the reaction produced the vinyldiboronates in a stereo- and chemoselective manner (Scheme 6).

In 2017, Santos et al. reported a substrate-assisted, transition-metal-free diboration reaction of alkynamides with Bpin–Bdan (Scheme 6) [29]. The installation of an amide directing group was used to control selectivity. The diboration transformation proceeded in a highly regio- and stereoselective fashion, and exclusively produced *trans*-vinyldiboronates. Bdan and Bpin were installed at the α- and β-carbon atoms of alkynamides, separately, in moderate to excellent yields, whose chemoselective arylation would produce densely-functionalized alkenes.

A density functional theory (DFT) study was performed to probe the reaction mechanism. Deprotonation of N–H in alkynamides with sodium hydride generated the charged Lewis base **A**, a naked anion with the metal counterion chelated by a crown ether. Thus, the difference in Lewis acidity of the two boron atoms in Bpin–Bdan could facilitate the selective complexation of base **A** to the Bpin moiety. With such an assistive effect, the transfer of the Bdan group to the α-carbon atom was favored (relative 0.0 kcal/mol), compared with the more energy-demanding β-carbon attack pathway (16.2 kcal/mol). Finally, an intramolecular bind between the resulting anion and Bpin produced the target product.

**Scheme 7:**
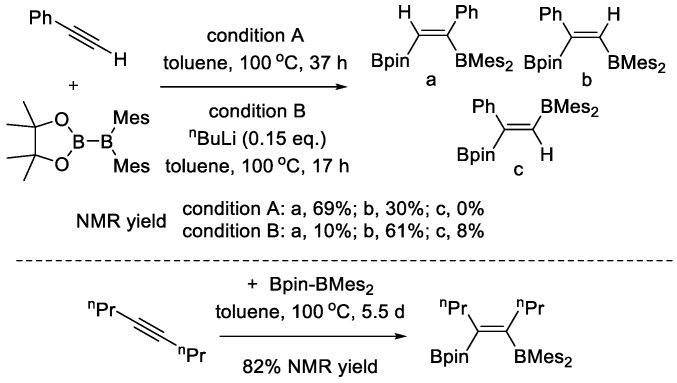
Bpin–BMes_2_ in the diboration of alkynes.

In addition to Bpin–Bdan, unsymmetrical Bpin–BMes_2_ could also be employed in the diboration of alkynes, which was disclosed by Yamashita et al. in 2016 [30]. A simple treatment of the aromatic and aliphatic terminal alkynes with Bpin–BMes_2_ in toluene resulted in two *cis*-isomers of diborated products. In the presence of a catalytic amount of ^n^BuLi, the selectivity was altered and *trans*-diboration products resulted. When internal alkynes were used, tetrasubstituted *syn*-diborylalkenes could be obtained, in spite of lower reaction rates (Scheme 7).

**Scheme 8:**
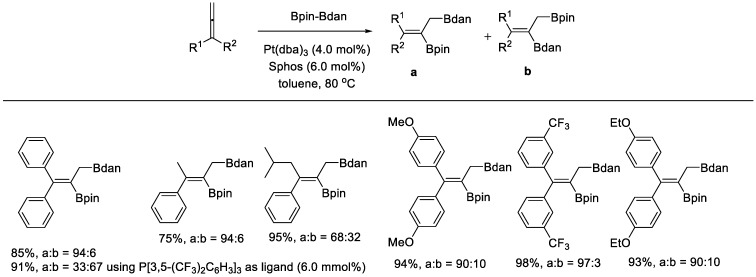
Pt-catalyzed diboration of allenes.

In 2015, Santos et al. disclosed a platinum-catalyzed regio- and chemoselective diboration of 1,1-disubstituted allenes, using Bpin–Bdan as the borylating reagent and producing vinyl and allyl boronates in good to excellent yields (Scheme 8) [31]. Chemoselective transfer of the Bpin moiety to the internal sp-hybridized carbon atom was observed. The Bdan moiety was incorporated into the terminal unsubstituted carbon atom. It should be noted that the ratios of several obtained isomers could be adjusted and even overturned, depending on the structure of the starting materials (a choice of catalyst and ligand combinations).

**Scheme 9:**
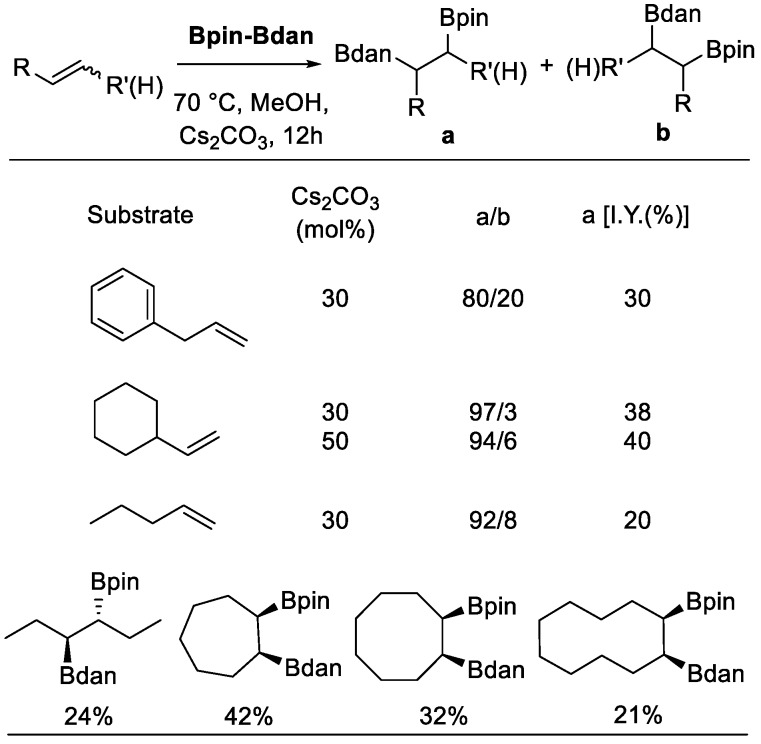
Diboration of alkenes.

In 2015, Fernández et al. reported a diboration reaction of olefins using Bpin–Bdan, under metal-free conditions (Scheme 9) [32]. Both terminal and internal alkenes were suitable starting materials. When the former type of substrates was used, the Bdan moiety was incorporated into the internal position with high regioselectivity preference. Mechanistically, Lewis acid-base adducts were formed in the beginning via the interaction between the alkoxide anion and the one boron atom of Bpin–Bdan to form the corresponding diborated alkenes. Due to the π-donation from the nitrogen lone pair to the boron orbital in the Bdan moiety, the boron atom of the Bpin moiety has a stronger Lewis acidity and, hence, a stronger affinity with alkoxide. Then, a selective nucleophile transfer was possible to deliver Bdan to the olefins. As for the substrate scope, allylbenzene, vinylcyclohexane, chain internal olefins, *cis*-3-hexene, and cyclic olefins were all suitable starting materials. Furthermore, the addition of cyclic olefins with Bpin and Bdan moieties took place in *syn* fashion. However, vinylarenes failed to undergo the diboration process.

**Scheme 10:**
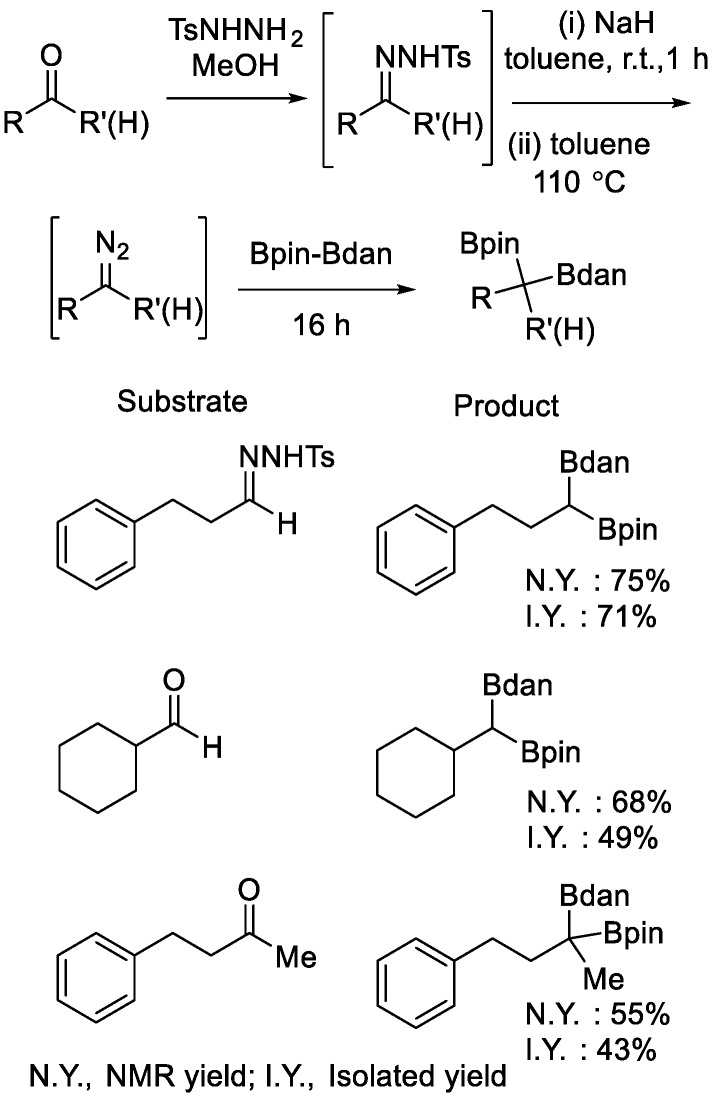
Diboration of aldehydes and ketones with Bpin–Bdan.

Fernández et al. also studied the diboration reaction of diazo compounds, which were prepared in situ from *N*-tosylhydrazones of aldehydes and ketones, using Bpin–Bdan [33]. After the metal-free process, the different boryl moieties of the mixed diboron reagent were both connected to the former sp^2^ carbon atoms of the diazo compounds, producing *gem*-diborated products (Scheme 10). A concerted, yet asynchronous reaction pathway was proposed based on DFT calculation.

**Scheme 11:**
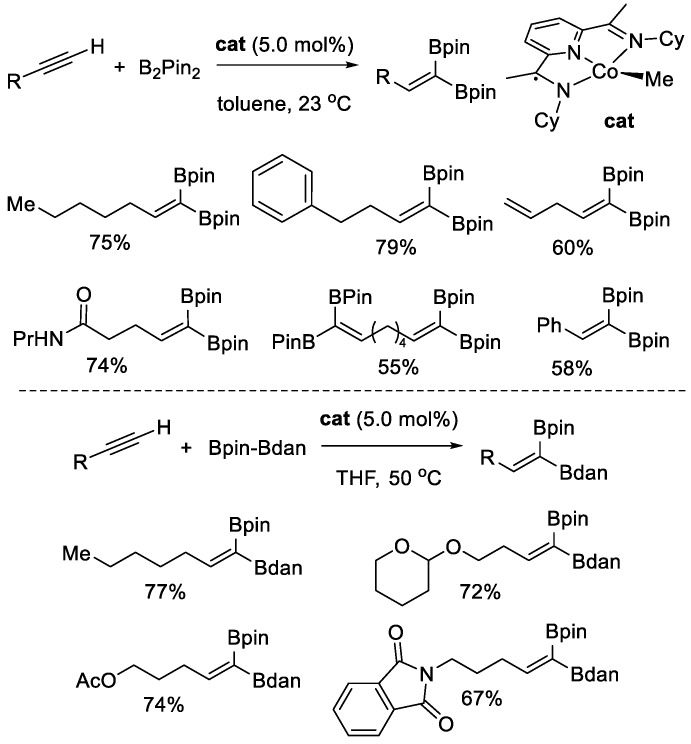
Cobalt-catalyzed 1,1-diboration of terminal alkynes.

In 2017, taking advantage of a cyclohexyl-substituted pyridine(diimine) cobalt methyl complex, Chirik et al. described the 1,1-diboration of readily available terminal alkynes using diboron reagents (Scheme 11) [34]. Such an addition reaction contrasted all other previously reported transition metal-catalyzed 1,2-diboration reactions. In this reaction, 1,1-Diboron olefins containing two identical or two different boryl groups could be obtained, using symmetrical (Bpin–Bpin) or unsymmetrical (Bpin–Bdan) diboron reagents separately. When using the mixed diboron reagent, the reactions proceeded efficiently and stereoselectively to produce trisubstituted olefins. The resultant chemically-differentiated boron substituents allowed for selective Suzuki-Miyaura coupling reactions, leaving Bdan intact.

### 3.2. Addition Reactions: Hydroboration

**Scheme 12:**
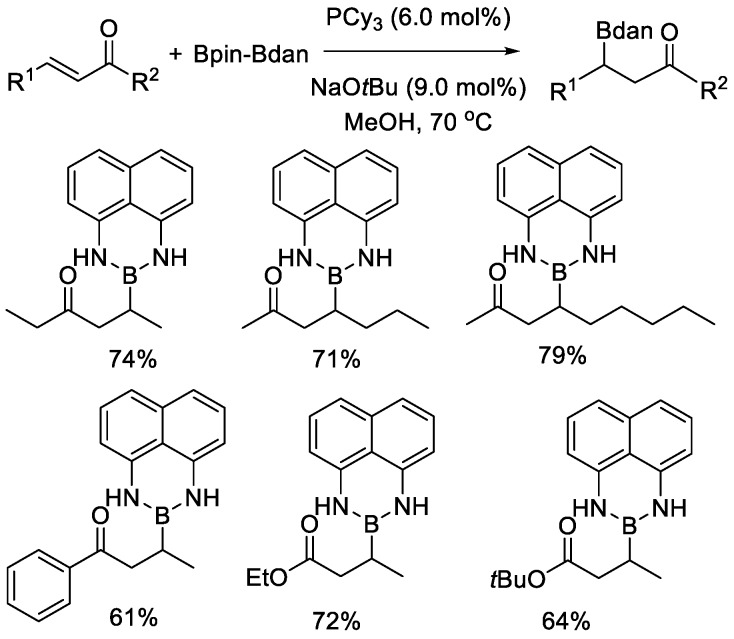
Organocatalytic β-boration of α,β-unsaturated carbonyl compounds with Bpin–Bdan.

Fernández, Carbó and Cid studied the application of Bpin–Bdan in organocatalytic β-boration of α,β-unsaturated carbonyl compounds in 2014 (Scheme 12) [35]. In the presence of a suitable base, selective activation of Bpin–Bdan with alkoxide was achieved, giving an MeO^−^→Bpin–Bdan adduct, which was demonstrated to be favorable compared with the MeO^−^→Bdan–Bpin adduct both experimentally and theoretically. Then, the Bdan moiety was directly transferred to the β-positions of the activated C–C bonds as a formal boron nucleophile with regioselectivity. This selectivity was also derived from the Lewis acidity difference of the two boron atoms, as mentioned above. The same strategy was then applied to the β-boration of α,β-unsaturated imines formed in situ [36]. An asymmetric version of this type of transformation was also described via the assistance of chiral phosphines.

Copper-catalyzed borylation reactions of unsaturated compounds, such as alkenes, alkynes, and arynes, have been well-developed as a relatively cheap method to incorporate boryl groups. A variety of reactions involving the addition of boryl groups, including hydroboration, diboration, borylstannylation, and carboboration, have been studied, which enabled a quick access to highly functionalized organoboron compounds.

**Scheme 13:**
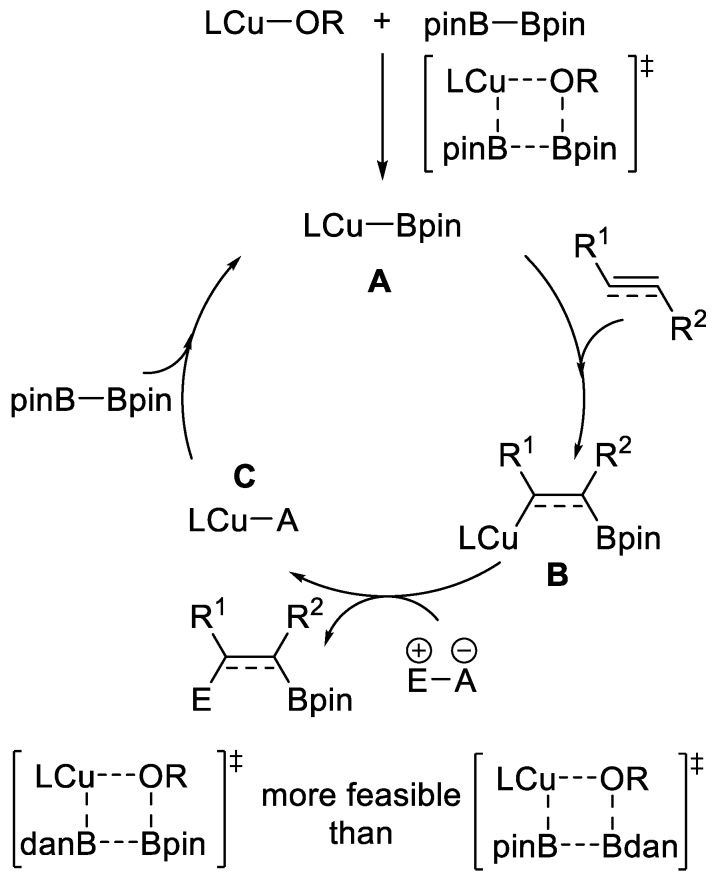
Mechanism of Cu-catalyzed boration of alkenes and alkynes.

Commonly, borylcopper species A, containing a Cu–B single bond, is considered to be the active catalyst in borylation reactions [12]. Subsequent insertion of a carbon-carbon triple or double bond into the Cu–B bond generates a β-boryl organocopper species B, which is followed by a reaction with an electrophile to produce the intermediate C, which then reacts with B_2_pin_2_ to accomplish the catalytic process. When Bpin–Bdan is used as the borylating reagent in such reactions, a Cu–Bdan species will be generated via σ-bond metathesis first, due to the selective interaction between the more Lewis acidic Bpin moiety and the alkoxy moiety of the precatalyst, Cu–OR. The transformation will add the Bdan unit to the unsaturated bonds (Scheme 13). More importantly, the Cu–Bdan species, in combination with suitable catalysts and ligands, is able to provide completely different regioselectivity compared with the traditional the Cu–Bpin species. This will be detailed hereinafter.

**Scheme 14:**
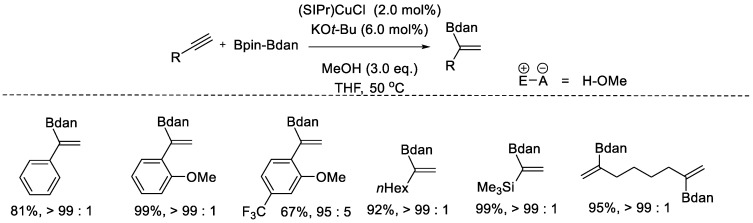
Cu-catalyzed hydroboration of terminal alkynes.

In 2014, Yoshida et al. developed the first general α-selective formal hydroboration of terminal alkynes (Scheme 14) [37]. Accordingly, diverse branched alkenylboron compounds were obtained exclusively. The use of Bpin–Bdan as the boryl source in the presence of a Cu–NHC (N-heterocyclic carbene) catalyst was essential to convert the common β-selectivity with B_2_pin_2_. Regardless of the electronic and steric nature of terminal alkynes employed, Markovnikov-type hydroboration products could be obtained in high yield and with high regioselectivity.

**Scheme 15:**
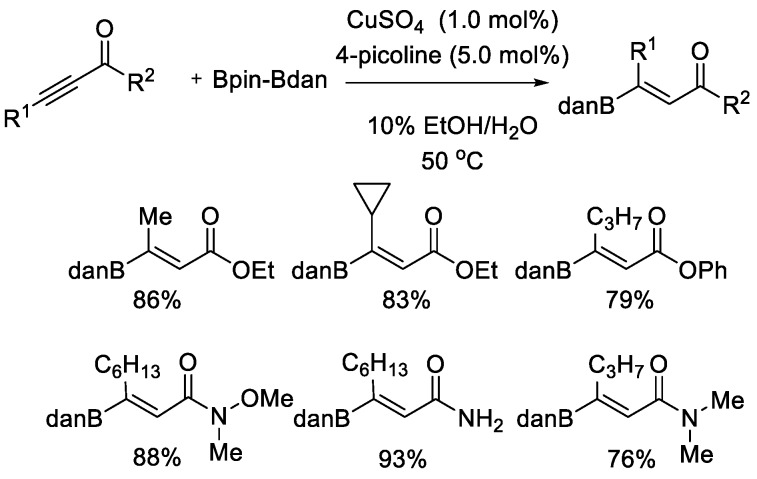
Cu-catalyzed hydroboration of alkynoates and alkynamides.

Later, another protocol for hydroboration of alkynes with Bpin–Bdan was disclosed by Santos et al. (Scheme 15) [38]. In this case, activated alkynes, such as alkynoates and alkynamides, were utilized. Aqueous and open-air conditions made this copper(II)-catalyzed process operationally feasible and simple. The reaction also proceeded in a remarkably chemo-, regio-, and stereoselective manner, producing (*Z*)-β-Bdan enoates and enamides in good-to-excellent yields.

### 3.3. Other Types of Addition Reactions

**Scheme 16:**
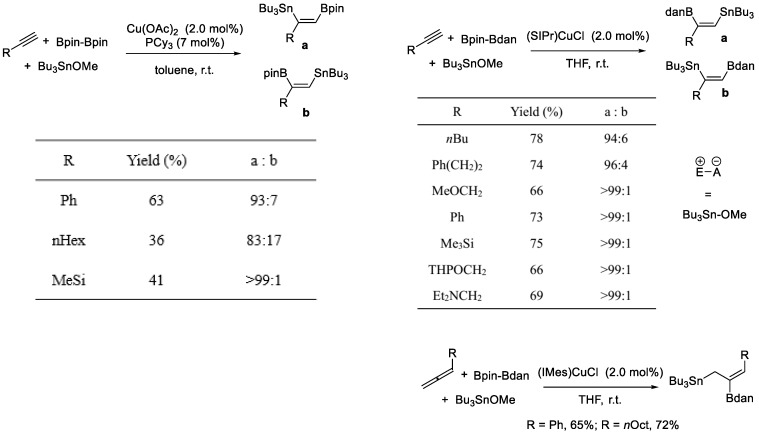
Cu-catalyzed borylstannylation of alkynes and allenes.

As mentioned above, the Cu–Bdan species could be added to terminal alkynes in a regioselective manner, which was inverse to the selectivity of the Cu–Bpin species. Taking advantage of this property, Yoshida et al. performed a Cu-catalyzed, three-component coupling reaction, which created borylstannylated products of alkynes (Scheme 16) [39]. Inverse regioselectivity was observed when using Bpin–Bdan, compared with B_2_pin_2_ [40]. Bu_3_SnOMe was used to supply the stannyl group and to capture the Cu–C bonds as an electrophile, as indicated in Scheme 13. A variety of *cis*-boryl(stannyl)alkenes could be easily prepared from terminal alkynes. In addition, allenes were also used as starting materials, and also underwent regio- and stereoselective borylstannylation reactions to provide (*Z*)-1-stannyl-2-boryl-2-alkenes. In this case, the Bdan moiety was transferred to the internal sp-hybridized carbon atom of allenes.

**Scheme 17:**
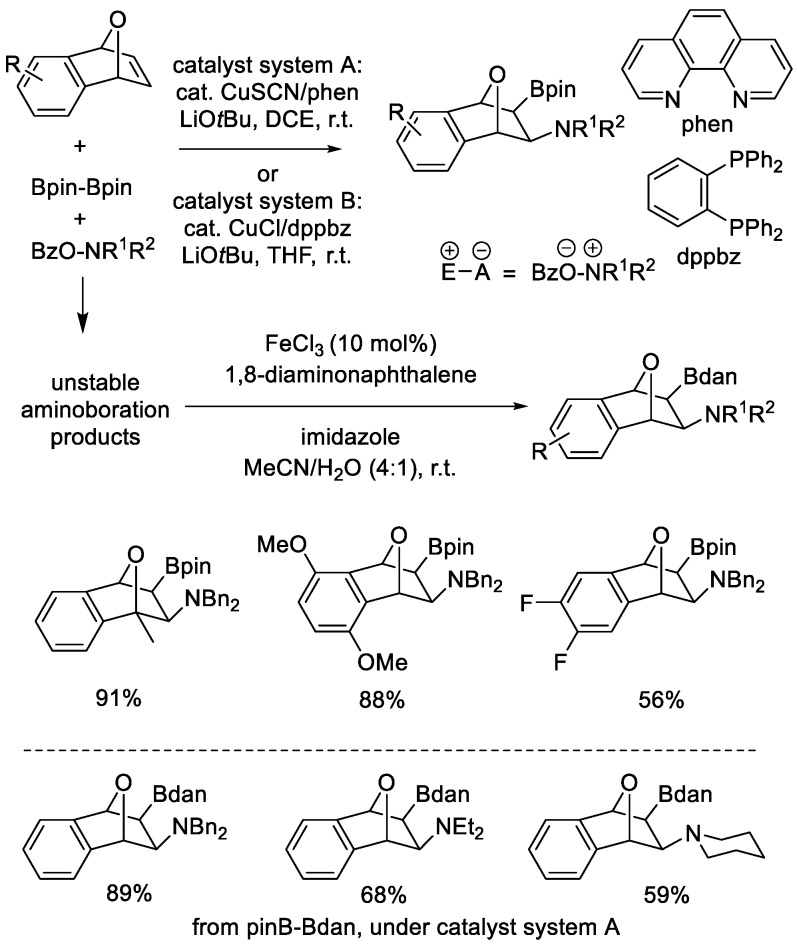
Cu-catalyzed aminoboration of bicyclic alkenes.

In 2014, Miura et al. performed a copper-catalyzed three-component reaction, which achieved the aminoboration of bicyclic alkenes with diboron reagents (B_2_pin_2_ or Bpin-Bdan) as the nucleophilic boron source and hydroxylamines as the amination reagent [41]. Two effective catalytic systems that promote this transformation were described after extensive screening of reaction variables without the formation of ring-opened byproducts. Various bicyclic alkenes, including oxa- and azabicycloalkenes, were suitable starting materials for this stereoselective aminoboration transformation. When B_2_pin_2_ was used, the difunctionalized products were less stable in several cases, which made the purification step problematic. To solve this problem, the crude materials were converted into the more stable Bdan derivatives, enabling the corresponding products to be isolated by column chromatography. Several examples that directly harnessed Bpin-Bdan as the borylating reagent were also explored. Similarly, the Bdan moiety was selectively transferred to alkenes (Scheme 17).

**Scheme 18:**
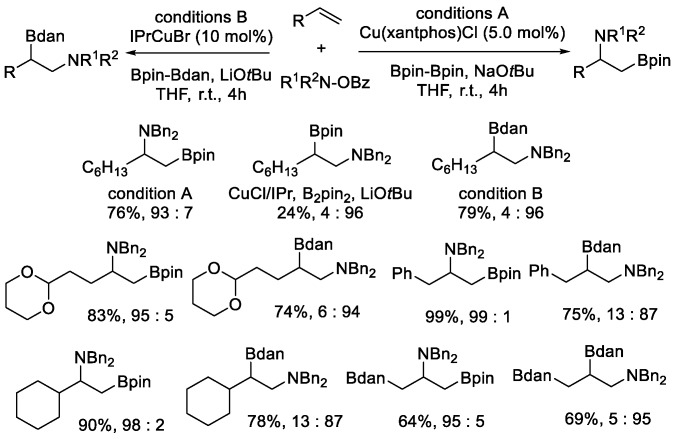
Regiodivergent Cu-catalyzed aminoboration of terminal alkenes.

Later, the same authors described a Cu-catalyzed aminoboration of unactivated terminal alkenes [42]. Interestingly, regioselectivity could be well regulated by a suitable combination of employed ligands and diboron reagents. According to the data on reaction condition screening, in the presence of B_2_pin_2_, the change of ligands alone could result in opposite regioselectivity. As indicated in Scheme 18, with isolated Cu(xantphos)Cl and NaO*t*Bu as the precatalyst and base, respectively, the terminally-borylated products have good yield with high regioselectivity (93:7). Conversely, a series of NHC ligands primarily led to the opposite regioisomer and IPr produced the highest regioselectivity (4:96). However, the yield of such an aminoboration reaction was poor (24%). When the borylating reagent was replaced with Bpin–Bdan, the reaction was improved significantly with a greatly increased yield and the maintenance of the unique regioselectivity. With these two conditions, a variety of valuable β-borylalkylamines were obtained regiodivergently.

**Scheme 19:**
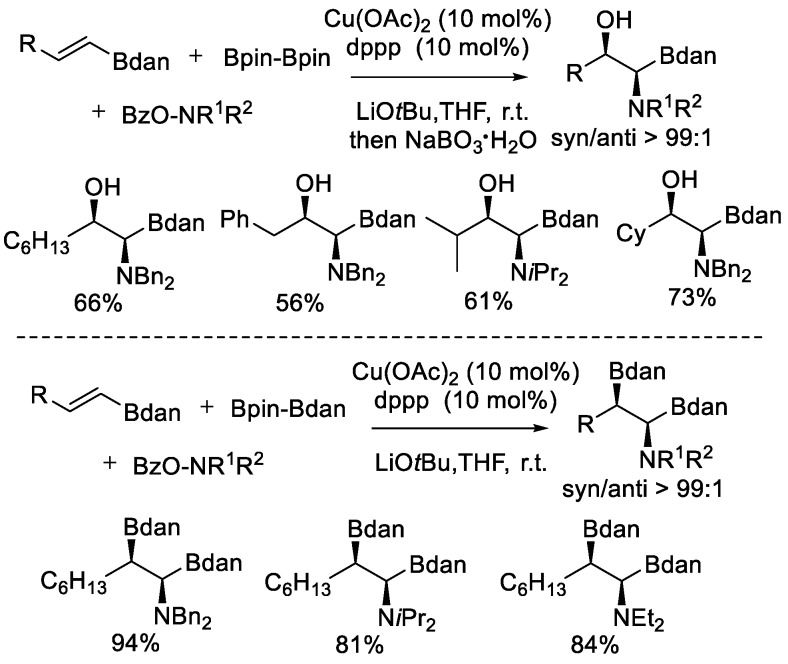
Cu-catalyzed aminoboration of alkenylboronates.

In 2016, another aminoboration of alkenes reaction was described by Miura et al. [43]. In this case, alkenyl 1,8-diaminonaphthyl (DAN) boronates were used as the starting materials (Scheme 19). The reaction proceeded in a regio- and stereoselective fashion to form the corresponding β-boryl-α-aminoboronic acid derivatives. The exploration began with (*E*)-2-octenyl DAN boronate, B_2_pin_2_, and *O*-benzoyl-*N*,*N*-dibenzylhydroxylamine. An extensive screening of reaction parameters demonstrated that the aminoboration proceeded smoothly at room temperature in the presence of a combination of Cu(OAc)_2_, 1,3-Bis(diphenylphosphino)propane (dppp), and LiO*t*Bu. A high *syn/anti* diastereoselectivtiy (99:1) was observed. Due to the instability of the obtained products, in situ oxidation by NaBO_3_·H_2_O was applied to facilitate the isolation of products. Another solution to solve the instability problem relied on the utilization of Bpin–Bdan. The standard Cu(OAc)_2_/dppp catalyst system used for B_2_pin_2_ was also suitable for Bpin–Bdan. Then, the more stable and easily handled Bdan moiety was introduced to the products, directly isolating the formed β-Bdan-α-aminoboronic DAN boronates.

**Scheme 20:**
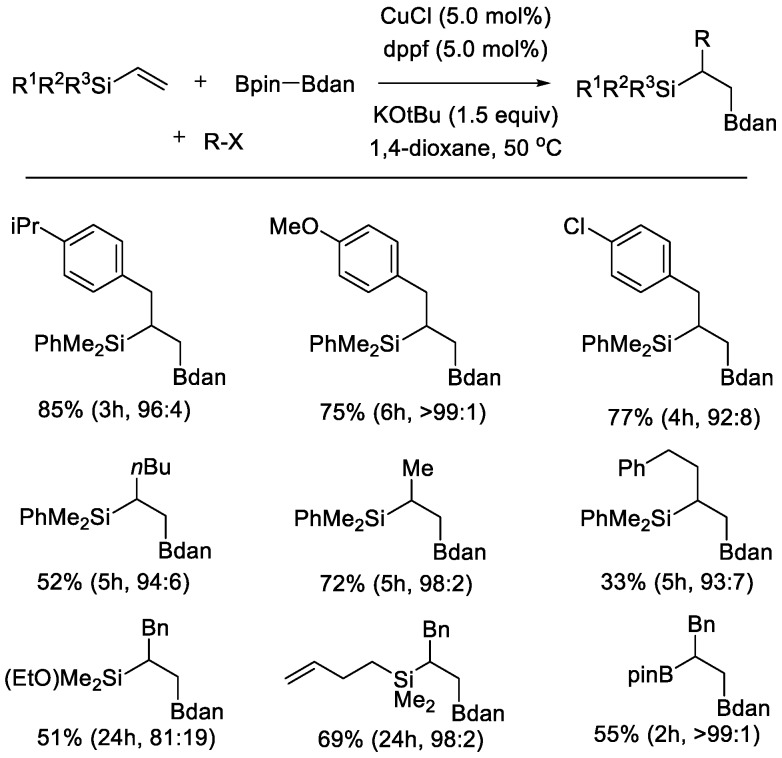
Cu-catalyzed carboboration of alkenes.

In 2017, Yoshida et al. described the first Cu-catalyzed Bdan-installing carboboration reaction using Bpin–Bdan [44]. Under CuCl/dppf (1,1′-Bis(diphenylphosphino)ferrocene) catalysis, a direct and regioselective approach to produce alkylboron compounds was established via three-component reactions (Scheme 20). As for the substrate scope, carbon-carbon double bonds attached to silicon atoms exhibited superior reactivity. Various substituted vinylsilanes underwent the carboboration reactions in a chemoselective and regioselective fashion. In addition, a vinylborane was also found to be a suitable starting material, and produced 1,2-diborylalkane bearing differentiated masked Bdan and Bpin boryl moieties.

## 4. Conclusions

Unsymmetrical diboron reagents, whether sp^2^-sp^3^ or sp^2^-sp^2^ hybridized, have been shown to be suitable borylating reagents in a variety of addition reactions, such as hydroboration and difunctionalization of alkenes or alkynes. Both metal-catalyzed and metal-free approaches for these transformations have been well established, offering diverse, densely functionalized alkyl and alkenyl boronic derivatives. Notably, the different regioselectivity of unsymmetrical diboron reagents compared with that of common symmetrical diboron reagents shows the potential of these borylating reactions to complement the existing borylating methods. In spite of these achievements, some problems still need to be addressed. First, the most commonly used Bpin–Bdan reagent usually transfers its Bdan to any unsaturated bonds. The obtained products will require additional deprotection steps to continue the transformation to relatively active boron species. Therefore, the development of the new masking groups is still very useful. In addition, the mechanism of the above-mentioned reactions still needs to be clarified, such as the driving force of the observed regioselectivity, and the cooperation and interaction of catalysts, ligands and substrates.
